# Cosmeceutical Potential of Extracts Derived from Fishery Industry Residues: Sardine Wastes and Codfish Frames

**DOI:** 10.3390/antiox11101925

**Published:** 2022-09-28

**Authors:** Martim Cardeira, Ana Bernardo, Inês C. Leonardo, Frédéric B. Gaspar, Marta Marques, Rodrigo Melgosa, Alexandre Paiva, Pedro Simões, Naiara Fernández, Ana Teresa Serra

**Affiliations:** 1 IBET—Instituto de Biologia Experimental e Tecnológica, Apartado 12, 2781-901 Oeiras, Portugal; 2 Instituto de Tecnologia Química e Biológica António Xavier, Universidade Nova de Lisboa (ITQB NOVA), Av. da República, 2780-157 Oeiras, Portugal; 3 LAQV-REQUIMTE—Associated Laboratory for Green Chemistry (LAQV) of the Network of Chemistry and Technology (REQUIMTE), Chemistry Department, NOVA School of Science and Technology, Universidade Nova de Lisboa, 2829-516 Caparica, Portugal

**Keywords:** fish waste streams valorisation, antioxidant activity, anti-inflammatory activity, antimicrobial activity, anti-ageing, anti-hyperpigmentation, cosmeceuticals

## Abstract

The fishery industry generates large amounts of waste (20–75% (*w*/*w*) of the total caught fish weight). The recovery of bioactive compounds from residues and their incorporation in cosmetics represents a promising market opportunity and may contribute to a sustainable valorisation of the sector. In this work, protein-rich extracts obtained by high-pressure technologies (supercritical CO_2_ and subcritical water) from sardine (*Sardina pilchardus*) waste and codfish (*Gadus morhua*) frames were characterized regarding their cosmeceutical potential. Antioxidant, anti-inflammatory and antibacterial activities were evaluated through chemical (ORAC assay), enzymatic (inhibition of elastase and tyrosinase), antimicrobial susceptibility (*Klebsiella pneumoniae*, *Staphylococcus aureus* and *Cutibacterium acnes*) and cell-based (in keratinocytes-HaCaT) assays. Sardine extracts presented the highest antibacterial activity, and the extract obtained using higher extraction temperatures (250 °C) and without the defatting step demonstrated the lowest minimum inhibitory concentration (MIC) values (1.17; 4.6; 0.59 mg/mL for *K. pneumoniae*, *S. aureus* and *C. acnes*, respectively). Codfish samples extracted at lower temperatures (90 °C) were the most effective anti-inflammatory agents (a concentration of 0.75 mg/mL reduced IL-8 and IL-6 levels by 58% and 47%, respectively, relative to the positive control). Threonine, valine, leucine, arginine and total protein content in the extracts were highlighted to present a high correlation with the reported bioactivities (R^2^ ≥ 0.7). These results support the potential application of extracts obtained from fishery industry wastes in cosmeceutical products with bioactive activities.

## 1. Introduction

With the constant search for innovation, especially for active ingredients, the cosmetic industry is growing and has demonstrated the intention to replace petroleum-derived components moving forward toward natural compounds [1]. The antioxidant properties of natural active ingredients can help in the prevention of several skin issues caused by oxidative stress and ageing [2,3]. Skin ageing can be induced by both intrinsic (such as inflammation or telomere shortening) and extrinsic (environmental) factors [4]. Skin ageing leads to the loss of mature collagen and alterations at the extracellular matrix (ECM) which compromises the barrier function, resulting in a dry appearance and susceptibility to external aggressors, increasing the risk for skin disorders [5]. This process can be accelerated by several enzymes, such as elastases, matrix metalloproteinases (MMPs) and hyaluronidases that can induce ECM degradation [6], or even by the accumulation of excessive reactive oxygen species (ROS) that can compromise the normal cell function [7]. Environmental factors, such as exposure to UV radiation, leads to the generation of high quantities of ROS that induces the same molecular and cellular responses as intrinsic ageing, but with amplified effects. Importantly, ROS can intensify the activity of enzymes related to skin ageing or skin pigmentation processes [8,9], and thus the presence of antioxidants can play an important role in the cosmetic field.

In 2018, world fish consumption was estimated by FAO to stand at 20.5 kg per capita [10], which leads to large quantities of by-products, mostly skin and bones. The generated residues correspond to 20–75% (*w*/*w*) of the total caught fish weight, potentially leading to environmental problems [11,12]. However, these residues still contain a significant amount of lipids, proteins, and minerals and should be adequately valorised. In recent years, extracts derived from waste generated by the fish industry have shown bioactive properties such as antihypertensive, antioxidative, antimicrobial, neuroprotective, antihyperglycemic, anti-ageing, and anti-inflammatory [13,14,15,16,17,18,19]. Atlantic codfish (*Gadus morhua*) and sardine (*Sardina pilchardus*) are among the most consumed fish in Portugal and extracts derived from its residues have shown promising nutraceutical potential, such as antioxidant, antiproliferative or anti-inflammatory activities [11,20,21,22]. However, since the exploitation and valorisation of fish industry wastes is still in an early stage, there is plenty of room to explore opportunities for the industry to convert this waste into high-value market bioproducts, including cosmetic ingredients.

In a previous work, we explored the use of high-pressure technologies (supercritical CO_2_ and subcritical water), to isolate bioactive fractions from sardine waste and codfish frames with promising health benefits [11,22]. For sardine wastes, we demonstrated that by applying a first step with supercritical carbon (ScCO_2_) (to remove lipid fraction) followed by an extraction process with subcritical water (SW) it was possible to obtain protein hydrolysates with high antioxidant potential and antiproliferative effect in colorectal cancer cells [22]. Subcritical water extraction/hydrolysis were also applied to obtain proteins-, peptides- and amino acid-enriched extracts from codfish frames and we showed that lower processing temperatures (90 °C) favour the extraction of compounds with anti-inflammatory potential in a human intestinal epithelial cell model [11]. Most of the proteins present in codfish frames extracts were collagen and collagen fragments. Other compounds include minor quantities of lipids, ash and some sugars. Sardine extracts were rich in peptides and amino acids, and lipids, ash and sugars were also present. Since fish-derived proteins and peptides may become an important resource for cosmetic industries, the present study aims to further evaluate the bioactive potential of these extracts derived from fish-processing wastes and by-products focused on the assessment of their cosmeceutical potential [23]. For this purpose, a range of chemical, enzymatic, and cell-based assays were applied to explore the antioxidant, anti-ageing, anti-hyperpigmentation, anti-inflammatory, and antimicrobial effects of the extracted samples. Correlation studies were also performed to identify the main bioactive constituents with cosmeceutical potential.

## 2. Materials and Methods

### 2.1. Reagents

3,4-dihydroxy-l-phenylalanine (L-DOPA), mushroom tyrosinase, porcine pancreatic elastase (PPE) type III, N-succinyl-Ala-Ala-Ala-p-nitroanilide (AAAPVN), Tris (2-amino-2-hydroxymethyl-propane-1,3-diol), 2,2′-azobis (2-methylpropionamidine)dihydrochlo-ride (AAPH), and 2′,7′-dichlorofluorescein diacetate (DCFH-DA) were purchased from Sigma-Aldrich (St. Louis, MO, USA). Calcium-adjusted Mueller Hinton broth (CAMHB) was purchased from BD (Sparks, MD, USA). Brain-heart infusion (BHI) was purchased from Avantor (Radnor, PA, USA). AnaeroGen™ Compact sachets were purchased from Oxoid (Hampshire, UK). PrestoBlue™, Dulbecco’s Modified Eagle Medium (DMEM), heat-inactivated Fetal Bovine Serum (FBS) and Penicillin-Streptomycin were obtained from Invitrogen (San Diego, CA, USA). Human immortalized non-tumorigenic keratinocyte cell line HaCaT was obtained from Cell Line Service (Eppelheim, Germany). Human IL-8 and IL-6 Mini TMB ELISA Development Kits were obtained from Peprotech (London, UK). All other reagents and solvents used in the present study were of analytical grade and purchased from available suppliers.

### 2.2. Samples

The extracts used in this work were the ones developed in our previous studies focused on process optimization [11,22]. Briefly, codfish frames were supplied by Pascoal and Filhos S.A. (Gafanha da Nazaré, Portugal) and consisted of fish backbone and adhered muscle. Sardine waste, made of heads, spines and viscera, was supplied by Conservas A Poveira S.A. (Póvoa de Varzim, Portugal). The proximate composition of the raw materials used have been presented in our earlier works [11,22]. Protein (47 wt %) and ash (39 wt %) were the major components of codfish frames, with small quantity of lipids and carbohydrates (2 wt % and 0.3 wt %, respectively). Collagen is found to be the major protein in codfish frames, and in this case, it accounts for ca. 65% of the total protein content of original waste. In contrast, sardine is an oily fish, thus its waste is much richer in lipids than codfish frames. Sardine wastes showed a lipid content of 26 wt % and a protein content of 52 wt %, the rest being ash (17 wt %) and carbohydrates (3 wt %).

The extracts from codfish frames (Cf1, Cf2, Cf3 and Cf4) and sardine wastes (S1, S2 and S3) selected for this work were obtained by high pressure technology in a lab-scale apparatus as previously described [11,22,24] using the conditions summarized in Table 1. Briefly, 60 g of ground codfish frames or sardine waste (defatted or non-defatted) were loaded into a high-pressure reactor that was put inside an oven. The water pump was switch on at desired flowrate (ca. 10 mL/min) and pressure was set to 100 bar. As soon as pressure reached that value, the electrical oven was switch on, and the experiment started. The different extracts were collected during 30 min at different temperatures (90–250 °C). S1 and S3 extracts were obtained after a defatting process of the sardine waste by ScCO_2_ before SW extraction. Subcritical water extraction experiments were duplicated. For each extract sample, 25 mL were taken in triplicate, lyophilized, and weighed to calculate the corresponding extraction yield. Analytical data—protein content—are expressed as mean ± standard deviation (SD) of triplicates. The information regarding the characterization of these extracts in terms of protein content, amino acid profile, major mineral compounds or toxic and heavy metals is described in our previous works [11,22].

Stock solutions of Cf1, Cf2, Cf3, Cf4, and S2 were prepared in Milli-Q H_2_O at a concentration of 100 mg/mL. The other samples, namely S1 and S3, were dissolved in DMSO (300 and 550 mg/mL, respectively) due to their lower solubility in water. Samples were frozen and kept at −20 °C until further use. For cellular assays, the samples were previously sterilized by heat (121 °C, 15 min) in an autoclave (Tuttnauer 3870 el, Breda, Netherlands).

### 2.3. Oxygen Radical Absorbance Capacity (ORAC) Assay

ORAC assay was performed to evaluate the antioxidant capacity of the samples towards peroxyl radicals (ROO^•^), following the method developed by Huang et al. [25], with some adjustments as reported previously [26]. Briefly, in a black 96-well microplate, 150 µL disodium fluorescein (0.3 μM) was added to 25 µL of sample dilutions and incubated for 10 min at 37 °C. Afterwards, the reaction was initiated by the addition of 25 μL of 2,2′-Azobis (2-amidinopropane) dihydrochloride (AAPH, 153 mM) and fluorescence (Ex/Em 485 ± 20/528 ± 20 nm) was measured for 40 min at 37 °C in a FLx800 fluorescence microplate reader (FL800 Bio-Tek Instruments, Winooski, VT, USA). A standard curve was prepared using 5, 10, 20, 30 and 40 μM of (6-hydroxy-2,5,7,8-tetramethylchroman-2-carboxylic acid (Trolox)). All solutions were prepared in phosphate-buffered saline (PBS), 75 mM, pH 7.4. The results are expressed as micromoles of Trolox equivalent antioxidant capacity per gram of extract (μ mol TEAC/g extract).

### 2.4. Enzymatic Assays

#### 2.4.1. Elastase Inhibition Assay

This assay was based on the work of Wittenauer et al. [27] with some modifications as described previously [6]. Elastase inhibitory activity is determined by a spectrophotometric method using porcine pancreatic elastase (PPE) and N-succinyl-Ala-Ala-Ala-p-nitroanilide (AAAPVN) as the enzyme-substrate, by monitoring the release of p-nitroaniline at 410 nm. PPE was dissolved in 100 mM Tris (2-amino-2-hydroxymethyl-propane-1,3-diol)-HCl buffer (pH = 8.0) to a concentration of 1 mg/mL and stored at 20 °C in aliquots. On the day of the assay, an aliquot was taken and diluted in buffer to a concentration of 0.03 U/mL, 10 µL was loaded in the wells of the microtiter plates together with 100 μL of the Tris-HCl buffer and 30 μL of each sample. After 20 min of pre-incubation at 25 °C, the reaction was initiated by the addition of 40 µL of the substrate AAAPVN (0.55 mM). Absorbance was measured for 20 min after the addition of AAAPVN at a BioTek Instruments EPOCH 2 spectrophotometer microplate reader. The calculations were made as described in Equation (1), where *A_control_* and *A_sample_* represent the absorbance at 410 nm in the absence or presence of the sample, respectively. Since DMSO was used to dissolve samples S1 and S3, this solvent was also tested and used as control for these samples. The potential of the extracts to inhibit elastase was evaluated with increasing concentrations, to determine dose-dependent relations and establish the half maximal inhibitory concentrations (IC_50_) values, indicating the capacity of each sample in enzymatic activity inhibition to an extent of 50%.

(1) $${\%}_{inhibition}=\frac{\left(A_{control}-A_{sample}\right)}{A_{control}}*100$$

All results are expressed as IC_50_ mean value with the lower and upper limits of a 95% confidence interval, obtained from at least three independent experiments.

#### 2.4.2. Tyrosinase Inhibition Assay

The tyrosinase inhibitory potential of the extracts was evaluated spectrophotometrically using mushroom tyrosinase and L-DOPA as the substrate [28]. Tyrosinase converts L-DOPA to dopaquinone, which will sequentially cyclize to form dopachrome. The dopachrome formation can be observed by measurement of the absorbance at 475 nm. The substrate was added to the enzyme in the presence of the sample dilutions, to a final concentration of 30 U/mL tyrosinase and 2.5 mM L-DOPA. Since DMSO was used to dissolve samples S1 and S3, this solvent was also tested and used as control for these samples. After incubation at 37 °C for 30 min, absorbance was measured at 475 nm on a BioTek Instruments EPOCH 2 microplate spectrophotometer. All the reagents were prepared in sodium phosphate buffer (SPB; 0.1 M, pH 6.8), prepared by mixing sodium phosphate dibasic dihydrate and sodium phosphate monobasic monohydrate, and the calculations were made as described in Equation (1). All results are expressed as IC_50_ mean value with the lower and upper limits of a 95% confidence interval, obtained from at least three independent experiments.

### 2.5. Antimicrobial Susceptibility Testing

The target microorganisms selected for the antibacterial activity assays were the gram-negative bacteria *Klebsiella pneumoniae* CECT 8453 and the gram-positive bacteria *Staphylococcus aureus* ATCC 6538 and *Cutibacterium acnes* ATCC 6919^T^. For *K. pneumoniae* CECT 8453 and *S. aureus* ATCC 6538, assays were performed according to the broth microdilution method of CLSI M07-A10 guidelines as previously described by Rodrigues et al. [29]. In short, extract stock solutions were distributed in a round bottom microtiter 96-well plate and 2-fold serially diluted in calcium-adjusted Mueller Hinton broth (CAMHB; BD, Sparks, MD, USA) to obtain a concentration range of solutions. The inoculum was prepared using the growth method to achieve a homogenous suspension in saline solution. The adjusted inoculum was additionally diluted in CAMHB to guarantee that, following inoculation, each well contained around 5 × 10^4^ CFU. The plates were incubated under aerobic conditions at 37 °C for 16 to 20 h. For *C. acnes*, the assays were performed as previously described with the use of brain-heart infusion (BHI) (Avantor, Radnor, PA, USA) broth instead of CAMHB and by incubating the microtiter plates for 70–74 h at 37 °C in anaerobic jars containing the atmosphere generation system AnaeroGen™ Compact (Oxoid, Hampshire, UK).

For each stock solution analysed, a positive control (CAMHB or BHI and diluted inoculum), a medium sterility control (uninoculated CAMHB or BHI), and an extract sterility control (uninoculated 2-fold extract stock solution in CAMHB or BHI) were performed accordingly. Minimum inhibitory concentration (MIC) values were the lowest concentration of a sample that visibly inhibited microbial growth after incubation. When needed, MIC values were confirmed using the cell viability reagent PrestoBlue™ (Invitrogen, San Diego, CA USA) following the manufacturer’s guidelines. An additional MIC value, designated MIC*, was also established and defined as the lowest concentration of a sample at which bacterial growth was visually and differentially affected in comparison to the positive control. Minimum bactericidal concentration (MBC) values were reported as the lowest concentration of a sample leading to at least 99.9% reduction in viable bacterial counts in comparison to the initial inoculum and for equal incubation time. Results were expressed as a median of the values obtained after three biological replicates. Since DMSO was used to dissolve samples S1 and S3, this solvent was also tested to ensure that the final concentration used did not interfere with the target microorganism, hence the assay.

### 2.6. Cell-Based Assays

#### 2.6.1. Cell Culture

Human keratinocyte cell line HaCaT (CLS, Germany) was cultured in a standard Dulbecco’s Modified Eagle Medium (DMEM) supplemented with 10% (*v*/*v*) fetal bovine serum (FBS) and 1% (*v*/*v*) penicillin-streptomycin. The cells were routinely maintained as monolayers in 75 cm^2^ culture flasks and incubated at 37 °C with 5% CO_2_ in a humidified atmosphere.

#### 2.6.2. In Vitro Cytotoxicity

Cytotoxicity assays were performed according to previous works [6]. Briefly, HaCaT cells were seeded at a density of 1.4 × 10^5^ cells/cm^2^ in 96 well plates. After 3 days, cells were incubated with different concentrations of each sample (Cf1; Cf2; Cf3; Cf4; S2—50, 25, 12.5, 6.25, 3.13, 1.56, 0.78, 0.39; S1—3, 1.5, 0.75, 0.38, 0.19, 0.09, 0.05, 0.02; S3—5.5, 2.75, 1.38, 0.69, 0.34, 0.17, 0.09, 0.04 mg/mL) diluted in culture medium (DMEM medium containing 0.5% FBS). Wells containing cells incubated only with culture medium supplemented with 0.5% (*v*/*v*) of FBS were used as control. Solvent controls with 50% water or 1% DMSO in culture medium were also performed to exclude solvent toxicity. After 24 h of incubation, the cell viability was evaluated using PrestoBlue^®^ (5% *v*/*v* in culture medium) for 2 h at 37 °C, 5% CO_2,_ according to the manufacturer’s instructions. After this, the fluorescence of each well was measured (Ex./Em. 560 ± 20/590 ± 20 nm) in an FLx800 fluorescence microplate reader (BioTek Instruments, Winooski, VT, USA). Cell viability was expressed as the percentage of viable cells relative to the control. Three independent experiments were performed in triplicate.

#### 2.6.3. Cellular Antioxidant Activity

Cellular antioxidant activity was evaluated following previously described methods [6,30], with some modifications. Briefly, HaCaT cells were seeded at a density of 1.4 × 10^5^ cells/cm^2^ in 96 well plates and the formation of intracellular ROS was monitored using 2′,7′-dichlorofluorescein diacetate (DCFH-DA) as a fluorescent probe. 72 h after seeding, cells were washed with PBS and incubated with non-toxic concentrations of the samples (0.1875 mg/mL; 0.375 mg/mL; 0.75 mg/mL) plus 25 µM DCFH-DA in PBS for 1 h. Subsequently, cells were washed again with PBS and incubated with the stress inducer (600 µM AAPH in PBS) for 1 h. After that, fluorescence was measured in an FL800 microplate fluorescence reader (Bio-Tek Instruments, Winooski, VT, USA) (Ex/Em 485 ± 20/528 ± 20 nm). The results are expressed as ROS percentage relative to the untreated control (cells treated with DCFH-DA and AAPH). Three independent experiments were performed in triplicate.

#### 2.6.4. Evaluation of IL-6 and IL-8 Secretion

Experiments were performed as previously described [31], with several modifications. Briefly, HaCaT cells were seeded at a density of 1 × 10^5^ cells/cm^2^ in 12 well plates. After 3 days, cells were stimulated with 15 μg/mL of lipopolysaccharides (LPS) from *Escherichia coli* and co-incubated with three different concentrations of each extract (0.1875 mg/mL; 0.375 mg/mL; 0.75 mg/mL) diluted in culture medium (DMEM medium containing 0.5% FBS). Cells incubated only with LPS and cells incubated with only culture media were used as positive and negative controls, respectively. After 24 h, supernatants were collected, centrifuged for 10 min at 2000 g and stored at −80 °C until further analysis. IL-6 and IL-8 levels were assessed by enzyme-linked immunosorbent assay (ELISA), using commercially available kits (PeproTech; London, UK), according to the manufacturer’s instructions, with absorbance measured at 450 nm with wavelength correction set at 620 nm in a microplate spectrophotometer (EPOCH 2, BioTek Instruments, Winooski, VT, USA). The results are expressed as IL-6 or IL-8 percentage relative to the positive control (cells stimulated with LPS). Three independent experiments were performed in triplicate.

### 2.7. Statistical Analysis

ORAC and cell-based assays results are expressed as the mean value ± SD, obtained from at least three independent experiments. For the enzymatic and cytotoxicity assays, the IC_50_ values were determined from dose-response curves through log10 plots using GraphPad Prism 8.4.3. software (GraphPad Software, Inc., La Jolla, CA, USA). Results are presented as IC_50_ with a 95% confidence interval. Statistical analysis of the results was performed using the former software. When homogeneous variance and a normal distribution of the data were verified, the results were analysed by one-way analysis of variance (ANOVA), followed by the Tukey test for multiple comparisons. In the case of heterogeneous variances or if the data were not normally distributed, an appropriate unpaired Student’s *t*-test was performed to determine whether the means were significantly different. A *p*-value ≤ 0.05 was accepted as statistically significant in all cases. Antimicrobial susceptibility testing results are expressed as the median value, obtained from at least three independent experiments.

## 3. Results and Discussion

This study aims to investigate the cosmeceutical potential of protein-rich extracts that were produced by high-pressure technologies from fishery industry wastes, namely sardine wastes and codfish frames [11,22]. The selection of extracts was based on previous results regarding their characterization and process conditions. For codfish, all extracts were selected aiming at evaluating the impact of the extraction temperature on the recovery of compounds with promising bioactive effects on the skin. For sardine extracts, only three extracts derived by both defatted and non-defatted raw materials, processed at higher temperatures (190 and 250 °C) and with the highest extraction yield (>45.7 g/100 g) were chosen. The results regarding the total protein content of each extract are presented in Table 1.

### 3.1. Antioxidant, Anti-Ageing and Anti-Hyperpigmentation Activities

The potential cosmeceutical effect of the extracts was initially screened using chemical and enzymatic assays to evaluate their antioxidant, anti-ageing, and anti-hyperpigmentation effect. For the antioxidant capacity, the ORAC assay was selected as it measures the ability of samples to scavenge biologically relevant ROS, namely peroxyl radicals, which are considered one of the main inducers of skin ageing [2,32]. The anti-ageing effect was also evaluated through elastase inhibition since this enzyme is reported to be responsible for the degradation of elastin and other ECM proteins [6]. For the anti-hyperpigmentation effect, the tyrosinase assay was used, to evaluate the capacity of samples to inhibit melanin production. Table 2 summarizes the ORAC and IC_50_ values of all extracts.

Our results show that sardine and codfish extracts presented antioxidant activity and inhibition effects on elastase and tyrosinase enzymes’ activity. Among sardine samples, S1 showed the highest ORAC value (1.94 ± 0.08 µmol TEAC/mg extract) followed by S3 and S2. These results are in accordance with a previous antioxidant evaluation through an alternative method (2,2-diphenyl-1-picrylhydrazyl-DPPH assay) where the extract S1 presented the lowest IC_50_ values [22]. The higher scavenging capacity towards peroxyl radicals of the S1 sample could be derived from peptides with different amino acid sequences present in the extracts since interactions among them can influence the radical scavenging ability [33]. Additionally, compounds generated in Maillard or other thermo-oxidation reactions might influence the antioxidant activity of the samples [34]. Despite the lowest ORAC value, samples S2 and S3 were the ones with the highest capacity in inhibiting tyrosinase and elastase activities, respectively.

Among codfish extracts, Cf4 was shown to have the highest antioxidant and anti-hyperpigmentation activities. SEC-GPC analysis of the extracts has shown that peptides of decreasing molecular weight were obtained when increasing the extraction temperature up to 250 °C [11]. The elastase inhibition capacity of this sample is within the range of values found for other Cf extracts, namely Cf1 and Cf3. However, for the concentrations tested, these two extracts showed no inhibition activity towards tyrosinase.

In the literature there are some reports showing that extracts from marine by-products presents relevant antioxidant activity and inhibition of tyrosinse. For instance, extracts derived from marine (*Scophthalmus maximus*) by-products by alkaline hydrolysis, have demonstrated antioxidant activity accessed by different chemical assays: 1,1-diphenyl-2-picrylhydrazyl (DPPH) radical-scavenging ability (36.12% in relation to control), ABTS (2,2′-azinobis-(3-ethyl-benzothiazoline-6-sulphonic acid) method (12.81 μg BHT/mL) and crocin bleaching assay (8.03 μg Trolox/mL) [35]. In another study, enzymatic extraction of alum-salted jellyfish (*Lobonema smithii*) showed to produce hydrolysates with high antioxidant activity (IC_50_ = 0.9 mg/mL for ABTS e DPPH assays) and tyrosinase inhibitory potential, with IC_50_ values ranging between 14.1 and 24.5 mg/mL [36], which are in the same order of magnitude as the ones obtained in this work. Additionally, extracts with similar antioxidant values (ORAC values – 0.4 to 3.5 µmol TEAC/mg extract) were obtained from another type of food industry residues, namely winemaking waste streams [6]. However, these winery residues extracts presented higher tyrosinase (IC_50_ from 4.0 to 0.14 mg extract/mL) and elastase (IC_50_ from 3.4 to 0.1 mg extract/mL) inhibitory capacities than those obtained in this work, probably due to the presence of phenolic compounds that are recognized to have several bioactivities. It is important to mention that in our study we used mushroom tyrosinase to screen the anti-hyperpigmentation effect of extracts, as this enzyme has been widely used in high throughput assays [37]. Nevertheless, since there are some controversies regarding the similarity and homology of this enzyme with mammalian/human tyrosinase [38,39,40], future studies involving mammalian cell lines should be considered to evaluate the potential anti-hyperpigmentation effect of sardine and codfish extracts.

To identify which compounds could be responsible for the bioactive response of extracts, correlation studies between bioactivity data and the extracts’ amino acid composition reported previously [11,22] were performed (Table S1). For antioxidant activity, the highest correlations (R^2^ ≥ 0.7) were obtained between ORAC value and total threonine, free valine, as well as free and total leucine for all extracts. In the case of sardine samples, a high correlation (R^2^ ≥ 0.94) was also obtained for free and total tryptophan content. Accordingly, all these amino acids have been previously reported to have antioxidant properties in several model systems [41,42,43]. For elastase and tyrosinase inhibition activities, the highest correlation coefficients (R^2^ ≥ 0.8) were obtained for total protein content and free arginine, respectively, suggesting that these compounds could have an important role in the potential anti-ageing and anti-hyperpigmentation effect of fish industry waste streams extracts.

### 3.2. Antibacterial Activity

*S. aureus* and *K. pneumoniae* were chosen as representative gram-positive and -negative bacteria to evaluate the antimicrobial capacity of the extracts. The antibacterial activity assays performed with codfish frame extracts revealed that all of them were able to affect both bacterial strains’ growth behaviour (MIC* results in Table 3 and Table 4). However, true growth inhibition (MIC values) did not occur in the presence of any of the extracts at the concentrations tested.

As S1 and S3 were solubilized in DMSO, assays were performed with this solvent to evaluate its influence on the results obtained for the sardine extracts. The results reveal that the maximum DMSO concentration used in the antibacterial activity testing of the different extracts (up to 12.5%) did not affect the growth of both bacterial strains.

In general, all sardine extracts inhibited the bacterial growth of both gram-positive and gram-negative selected strains, but *S. aureus* was shown to be more susceptible than *K. pneumoniae*, which is expected since the outer membrane of gram-negative bacteria poses an additional barrier to prevent the interference of different molecules with the cell [44]. S2 was shown to be the extract with the most promising antibacterial potential, indicating that higher extraction temperatures favoured the extraction of anti-bacterial compounds. Taking these results into account, S2 was selected to be tested against another gram-positive bacteria, namely *C. acnes*, an aerotolerant anaerobe linked to the acne skin condition [45]. To evaluate the impact of the defatting process on the inhibition capacity of *C. acnes* growth, sample S3 was also selected to be tested in this assay. The results are summarized in Table 5 showing that *C. acnes* behaves similarly to *S. aureus* with its growth being affected by both sardine extracts. Between both samples, S2 presented the highest inhibitory effect (lower MIC and MBC values) suggesting that the defatting process could modify or eliminate some anti-microbial compounds from the sardine samples before the extraction process, especially free fatty acids and lipid oxidation products [22].

Previous studies showed that some amino acid residues present in peptides can lead to different antibacterial activities [46]. Rodrigues et al. showed that protein derivative-rich extracts obtained by extraction with deep eutectic solvents (DES) from sardine processing waste streams have antibacterial activity toward *S. aureus* and *Escherichia coli* [47]. However, the MIC*, MIC and MBC values of these extracts were lower than those obtained in this work for the same raw material, which could be explained by the synergic and/or additive effect between the extracts’ components and DES, as described previously [29]. Nevertheless, the antimicrobial effect of S2 extract is similar to other extracts obtained by subcritical water extraction, namely using kānuka leaves (*S. aureus*—MIC: 0.9 mg/mL, MBC: 3.8–5 mg/mL; *E. coli*—MIC: 3.8–7.5 mg/mL, MBC: 4.4–7.5 mg/mL) [48], that are rich in phenolic compounds already recognized as presenting relevant antimicrobial effects [49].

In this study, the highest correlation coefficient obtained was for MIC values of *S. aureus* and the total glutamic acid content (R^2^ = 0.6, Table S1).

### 3.3. Cellular Antioxidant and Anti-Inflammatory Effect

In this work, a human keratinocyte cell line (HaCaT) was used to better understand the bioactivity, namely antioxidant and anti-inflammatory effects, of extracts as some of the processes related to the uptake, distribution, and metabolism of bioactive compounds are better addressed [50]. HaCaT cells are one of the predominant cell types encountered in the skin, being responsible for skin integrity and, when affected by senescence or oxidative stress, can accelerate the skin ageing process [51]. Additionally, keratinocytes play an important role in the regulation of skin inflammation, responding to external stimuli, such as bacterial LPS, actively contributing to inflammation pathways especially by releasing proinflammatory cytokines or chemokines [31,52].

In a first approach, cytotoxicity assays were performed to evaluate the safety of samples and to select non-toxic concentrations for further bioactivity studies. Table 6 presents the IC_50_ values of each sample showing that S1 presented the highest cytotoxic effect. In previous studies, these samples showed higher IC_50_ values in Caco-2, a model for crypt enterocytes, than in HaCaT, which indicates that the samples are more toxic for keratinocytes than intestinal cells [11,22].

**Figure 1:**
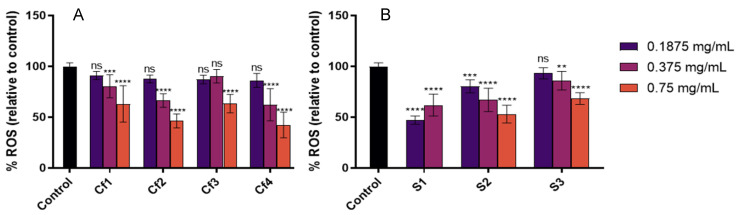
Cellular antioxidant capacity, expressed as % of ROS inhibition relative to the control, of each extract. (**A**) Codfish frame extracts; (**B**) Sardine wastes extracts. The results are expressed as mean ROS percentage relative to the control ± SD. The symbol * indicates significance relative to the control ** *p*-value ≤ 0.01, *** *p*-value ≤ 0.001, **** *p*-value ≤ 0.0001); ns—not significant.

The cellular antioxidant activity was then assessed by evaluating the capacity of samples in scavenging intracellular ROS generated by the chemical stressor AAPH. In parallel, anti-inflammatory assays were also performed to investigate the effect of samples in reducing the secretion of IL-8, which has been consistently reported as an important skin inflammation biomarker [53,54,55], upon pro-inflammatory stimulus with LPS. In these assays, non-cytotoxic concentrations of the extracts were used (0.1875, 0.375 and 0.75 mg/mL for all samples except S1 where 0.75 mg/mL was not tested since this concentration presented a cytotoxic effect, Table 6). In general, all samples inhibited ROS formation in HaCaT, and a dose-dependent effect was observed (Figure 1). Among codfish samples, Cf2 and Cf4 showed the highest cellular antioxidant activities, and amongst sardine samples, S1 showed the highest ROS percentage reduction relative to control.

**Figure 2:**
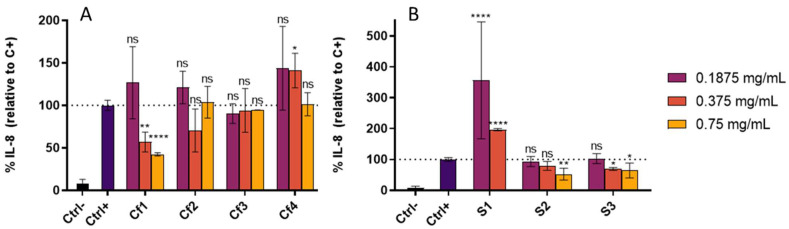
IL-8 secreted by HaCaT cells treated for 24 h with different extracts concentrations and 15 µg/mL LPS. (**A**) Codfish residues extracts; (**B**) Sardine residues extracts. Ctrl-—cells incubated with only culture media; Ctrl+—Cells incubated with culture media + inflammation inductor (LPS); Cf1, Cf2, Cf3, Cf4—cells incubated with the different extracts from codfish frames + inflammation inductor (LPS); S1, S2, S3—cells incubated with the different extracts from sardine wastes + inflammation inductor (LPS). The results are expressed as mean IL-8 percentage relative to the positive control ± SD. The symbol * indicates significance relative to the positive control (* *p*-value ≤ 0.05, ** *p*-value ≤ 0.01, **** *p*-value ≤ 0.0001); ns—not significant.

Concerning the anti-inflammatory effect, only Cf1 (0.375 and 0.75 mg/mL), S2 (0.75 mg/mL) and S3 (0.375 and 0.75 mg/mL) revealed capacity to inhibit IL-8 secretion by HaCaT cells after LPS-induced inflammation (Figure 2). In contrast, the other samples did not reduce IL-8 and, for some concentrations, Cf4 and S1 showed a pro-inflammatory effect. These two extracts were the ones that demonstrated the highest cytotoxic effect in HaCaT cells and thus the concentrations tested, although not leading to cell death, could induce the activation of inflammatory pathways since injured cells can release danger signals that alert other cells to cell death [56].

**Figure 3:**
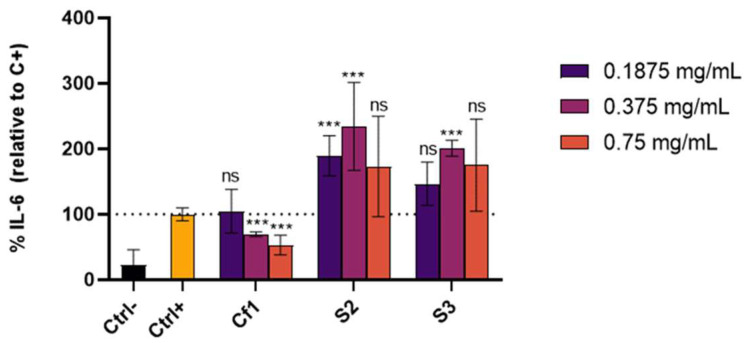
IL-6 secreted by HaCaT cells treated for 24 h with different extracts concentrations and 15 µg/mL LPS. Ctrl-—cells incubated with only culture media; Ctrl+—Cells incubated with culture media + inflammation inductor (LPS); Cf1– cells incubated with Cf1 extract from codfish frames + inflammation inductor (LPS); S2, S3—cells incubated S2 and S3 extracts from sardine wastes + inflammation inductor (LPS). The results are expressed as mean IL-6 percentage relatively to the positive control ± SD. The symbol * indicates significance relative to the positive control (*** *p*-value ≤ 0.001); ns—not significant.

The extracts with the highest anti-inflammatory effect, namely Cf1, S2 and S3, were further selected to evaluate their capacity in inhibiting IL-6 secretion, a cytokine also recognized as an important biomarker in skin disorders [53]. Results show that Cf1 was the only sample able to significantly reduce IL-6 secretion by 69.4 ± 3.5 and 53.0 ± 14.7% IL-6 (relative to the positive control, *p*-value < 0.001) at 0.375 and 0.75 mg/mL, respectively, whereas sardine extracts increased in IL-6 levels of the supernatants (Figure 3). Overall, only Cf1 revealed the capacity to reduce both IL-8 and IL-6 secretion by HaCaT cells, suggesting that this codfish extract could be further explored for anti-inflammatory applications in skin conditions.

The anti-inflammatory activity of codfish extracts was previously evaluated in an intestinal cell line (Caco-2 cells) [11] and the results were in line with the data presented in this study for HaCaT cells. In both cell lines, Cf1 presented the highest anti-inflammatory effect, reinforcing the use of lower temperatures to extract bioactive compounds from codfish frames.

Our results are in accordance with previous studies supporting the idea that fish-derived extracts/hydrolysates display a broad spectrum of bioactivities, including antioxidant, antimicrobial, anti-ageing, anti-hypertensive, anti-human immunodeficiency virus, anti-proliferative, or anticoagulant activities [23,57]. Song et al. showed that enzymatic hydrolysates of the marine fish half-fin anchovy contained antibacterial peptide fractions, with activity against *E. coli* [58]. Fish skin and collagen hydrolysed by subcritical water hydrolysis also showed high antioxidant and antimicrobial activity against *Bacillus cereus*, *S. aureus* and *Pseudomonas putida* [59]. Additionally, Wang and co-workers produced extracts derived from fish side streams of two fish species (rainbow trout and sole) that could inhibit the growth of pathogenic bacteria *(S. aureus* or *Salmonella*) and with anti-inflammatory properties [60].

## 4. Conclusions

In this work, we applied a platform of in vitro bioassays to evaluate for the first time the cosmeceutical potential of protein extracts derived from fishery wastes, namely sardine residues and codfish frames, obtained by high-pressure technology. We demonstrated that both types of extracts showed antioxidant, anti-ageing, and anti-hyperpigmentation potential. Among all, sardine extracts presented the highest anti-bacterial activity, and this effect was more pronounced for samples obtained using higher extraction temperatures (250 °C) and without the defatting step. Codfish samples were the most effective anti-inflammatory agents, and in this case, lower temperatures (90 °C) favoured the extraction of these bioactive compounds. Although further studies are needed to identify which compounds could be responsible for the bioactive effects, total threonine, free valine as well as free and total leucine were identified to highly correlate with the antioxidant activities of samples. Total protein content and free arginine correlated with elastase and tyrosinase inhibition activities, respectively.

This work is a step forward in the development of potential cosmeceutical ingredients with antioxidant, skin whitening, antimicrobial, and anti-inflammatory effects from sardine residues and codfish frames, adding potential high value to these fishery industry wastes.

## Figures and Tables

**Table 1:** Extraction process techniques and parameters used for each sample. Extraction yield and protein content are expressed as mean ± standard deviation (SD).

Sample	Defatting Conditions	SW Extraction Conditions	Extraction Yield (g/100 g Feed) [11,22]	Protein Content (wt %) [11,22]
Cf1	-	90 °C, 100 bar	13.2 ± 0.5	81.6 ± 0.3
Cf2	-	140 °C, 100 bar	27.7 ± 0.5	93.6 ± 0.3
Cf3	-	190 °C, 100 bar	41.4 ± 0.5	95 ± 0.3
Cf4	-	250 °C, 100 bar	53.9 ± 0.5	84.4 ± 0.3
S1	ScCO_2_ (40 °C, 250 bar)	190 °C, 100 bar	45.7 ± 2.8	87.5 ± 2.7
S2	-	250 °C, 100 bar	58.5 ± 0.4	57.5 ± 1.8
S3	ScCO_2_ (40 °C, 250 bar)	250 °C, 100 bar	61.7 ± 2.0	85.2 ± 0.6

Cf1, Cf2, Cf3, Cf4—extracts from codfish frames; S1, S2, S3—extracts from sardine wastes.

**Table 2:** Antioxidant, anti-ageing, and anti-hyperpigmentation activities of codfish and sardine residues extracts. ORAC values are expressed as the mean value ± SD. Enzymatic assays IC_50_ values are expressed as mean with a 95% confidence interval.

Sample	Antioxidant Activity	Anti-Ageing Activity	Anti-Hyperpigmentation Activity
	ORAC	Elastase Inhibition	Tyrosinase Inhibition
	(µmol TEAC/mg extract)	(IC_50_, mg extract/mL)	(IC_50_, mg extract/mL)
Cf1	0.64 ± 0.18	42.58 (37.38, 49.88)	>100 **
Cf2	0.54 ± 0.22	60.35 (50.71, 73.43)	78.59 (61.41, 100.70)
Cf3	0.59 ± 0.26	34.74 (24.57, 49.16)	>100 **
Cf4	1.29 ± 0.26	38.11 (30.42, 49.52)	40.89 (30.26, 54.43)
S1	1.94 ± 0.08	44.29 (39.10, 53.44)	82.51 (57.12, 108.39)
S2	1.19 ± 0.10	>300 *	3.70 (3.26, 4.42)
S3	1.24 ± 0.06	17.96 (12.36, 25.83)	10.40 (5.02, 18.15)

* The maximum concentration tested was 300 mg/mL; ** The maximum concentration tested was 100 mg/mL.

**Table 3:** Antibacterial activity of codfish and sardine extracts against *S. aureus*.

Sample	MIC* Median	MIC Median	MBC Median
	(mg/mL)	(mg/mL)	(mg/mL)
	(n = 1/n = 2/n = 3)	(n = 1/n = 2/n = 3)	(n = 1/n = 2/n = 3)
Cf1	0.39	>50.00	>50.00
	(0.39/0.39/0.20)	(>50.00/>50.00/>50.00)	(>50.00/>50.00/>50.00)
Cf2	0.78	>50.00	>50.00
	(0.78/0.78/0.39)	(>50.00/>50.00/>50.00)	(>50.00/>50.00/>50.00)
Cf3	0.39	>50.00	>50.00
	(0.39/0.39/0.20)	(>50.00/>50.00/>50.00)	(>50.00/>50.00/>50.00)
Cf4	0.10	>50.00	>50.00
	(0.10/0.10/0.10)	(>50.00/>50.00/>50.00)	(>50.00/>50.00/>50.00)
S1	1.56	25.00	50.00
	(1.56/1.56/0.78)	(25.00/25.00/25.00)	(50.00/50.00/50.00)
S2	0.07	1.17	9.38
	(0.07/0.07/0.07)	(1.17/2.34/1.17)	(9.38/9.38/9.38)
S3	0.27	68.75	68.75
	(0.27/0.54/0.27)	(68.75/68.75/34.38)	(68.75/68.75/68.75)

MIC*—Lowest concentration of a sample at which bacterial growth was visually and differentially affected; MIC—Minimum inhibitory concentration; MBC—Minimum bactericidal concentration.

**Table 4:** Antibacterial activity of codfish and sardine extracts against *K. pneumoniae*.

Sample	MIC* Median	MIC Median	MBC Median
	(mg/mL)	(mg/mL)	(mg/mL)
	(n = 1/n = 2/n = 3)	(n = 1/n = 2/n = 3)	(n = 1/n = 2/n = 3)
Cf1	12.50	>50.00	> 50.00
	(12.50/12.50/12.50)	(>50.00/>50.00/>50.00)	(>50.00/>50.00/>50.00)
Cf2	25.00	>50.00	>50.00
	(25.00/25.00/50.00)	(>50.00/>50.00/>50.00)	(>50.00/>50.00/>50.00)
Cf3	0.78	>50.00	>50.00
	(0.39/0.78/0.78)	(>50.00/>50.00/>50.00)	(>50.00/>50.00/>50.00)
Cf4	0.39	>50.00	>50.00
	(0.39/0.39/0.20)	(>50.00/>50.00/>50.00)	(>50.00/>50.00/>50.00)
S1	3.13	50.00	50.00
	(3.13/3.13/6.25)	(50.00/50.00/50.00)	(50.00/50.00/50.00)
S2	0.07	4.69	18.75
	(0.07/0.07/0.07)	(4.69/4.69/9.38)	(18.75/18.75/18.75)
S3	0.54	68.75	68.75
	(0.54/1.07/0.54)	(68.75/68.75/68.75)	(68.75/>68.75/68.75)

MIC*—Lowest concentration of a sample at which bacterial growth was visually and differentially affected; MIC—Minimum inhibitory concentration; MBC—Minimum bactericidal concentration.

**Table 5:** Antibacterial activity of sardine extracts against *C. acnes*.

Sample	MIC* Median	MIC Median	MBC Median
	(mg/mL)	(mg/mL)	(mg/mL)
	(n = 1/n = 2/n = 3)	(n = 1/n = 2/n = 3)	(n = 1/n = 2/n = 3)
S2	0.29	0.59	2.34
	(0.29/0.29/0.29)	(0.59/0.29/0.59)	(4.69/1.17/2.34)
S3	2.15	17.19	> 68.75
	(2.15/2.15/2.15)	(34.38/17.19/17.19)	(>68.75/68.75/>68.75)

MIC*—Lowest concentration of a sample at which bacterial growth was visually and differentially affected; MIC—Minimum inhibitory concentration; MBC—Minimum bactericidal concentration.

**Table 6:** IC_50_ values obtained for all the extracts in HaCaT cells, with 24 h incubation. IC_50_ values are expressed as mean with a 95% confidence interval.

Sample	IC_50_ (mg Extract/mL)
Cf1	9.7 (9.5, 9.9)
Cf2	3.6 (3.5, 3.7)
Cf3	17.3 (15.2, 19.6)
Cf4	2.0 (1.9, 2.2)
S1	0.6 (0.5, 0.7)
S2	3.4 (3.3, 3.6)
S3	>5.5

IC_50_—concentration of the sample that leads to a decrease of 50 % of the cell population after 24 h incubation.

## Data Availability

The data supporting the findings of this study are available within the article and its supplementary material.

## Supplementary Materials

The following supporting information can be downloaded at: https://www.mdpi.com/article/10.3390/antiox11101925/s1, Table S1: Correlation coefficients between protein and amino acids content and different bioactivities of sardine waste and codfish frames extracts.
