# Supplementary material for: Cosmeceutical Potential of Extracts Derived from Fishery Industry Residues: Sardine Wastes and Codfish Frames
Source: Antioxidants (Basel). 2022 Sep 28;11(10):1925. doi: 10.3390/antiox11101925 (PMC9598070; doi:10.3390/antiox11101925)
Supplement: Supplementary file 1 [file antioxidants-11-01925-s001.zip › antioxidants-1869621-supplementary.pdf]

Supplementary Material

Cosmeceutical potential of extracts derived from fishery industry residues: sardine wastes and codfish frames

Martim Cardeira, Ana Bernardo, Inês C. Leonardo, Frédéric B. Gaspar, Marta Marques, Rodrigo Melgosa, Alexandre Paiva, Pedro Simões, Naiara Fernández, Ana Teresa Serra

Table S1 - Correlation coefficients between protein and amino acids content and different bioactivities of sardine waste and codfish frames extracts

		Ela	Tyr	*S. aureus*			*K. pneumoniae*			ORAC	CV	CAA		IL8	
				MIC*	MIC	MBC	MIC*	MIC	MBC			0.19 mg/mL	0.38 mg/mL	0.19 mg/mL	0.38 mg/mL
**Protein**		0.803	0.437	0.178	0.530	0.657	0.123	0.682	0.586	0.059	0.026	0.000	0.061	0.033	0.027
**Arg**	**Free**	0.849	0.018	0.165	nd	nd	0.120	nd	nd	0.619	0.323	0.775	0.498	0.182	0.827
	**Total**	0.493	0.066	0.042	nd	nd	0.279	nd	nd	0.142	0.955	0.038	0.928	0.608	0.035
**Lys**	**Free**	0.353	0.195	0.146	0.050	0.140	0.000	0.142	0.131	0.007	0.083	0.026	0.356	0.062	0.003
	**Total**	0.519	0.050	0.010	0.208	0.062	0.407	0.075	0.034	0.659	0.063	0.220	0.049	0.117	0.130
**Ala**	**Free**	0.271	0.011	0.169	0.006	0.017	0.218	0.017	0.015	0.180	0.034	0.001	0.234	0.000	0.129
	**Total**	0.598	0.161	0.002	0.283	0.136	0.327	0.155	0.091	0.524	0.042	0.175	0.064	0.058	0.078
**Thr**	**Free**	0.009	0.179	0.214	0.027	0.004	0.180	0.005	0.002	0.000	0.002	0.064	0.034	0.019	0.001
	**Total**	0.156	0.118	0.166	0.006	0.062	0.331	0.055	0.081	0.864	0.071	0.382	0.068	0.426	0.476
**Gly**	**Free**	0.185	0.026	0.151	0.000	0.003	0.225	0.003	0.004	0.130	0.032	0.002	0.187	0.000	0.163
	**Total**	0.346	0.479	0.001	0.398	0.305	0.055	0.331	0.237	0.116	0.026	0.070	0.004	0.001	0.014
**Val**	**Free**	0.000	0.041	0.514	0.238	0.012	0.095	0.010	0.015	0.788	0.158	0.925	0.425	0.848	0.869
	**Total**	0.111	0.019	0.273	0.013	0.045	0.105	0.037	0.069	0.562	0.167	0.334	0.005	0.339	0.159
**Ser**	**Free**	0.250	0.410	0.020	0.410	0.269	0.124	0.289	0.217	0.155	0.013	0.158	0.005	0.013	0.008
	**Total**	0.144	0.095	0.127	0.187	0.049	0.003	0.058	0.029	0.248	0.034	0.189	0.019	0.134	0.001
**Pro**	**Free**	0.061	0.034	0.263	0.104	0.023	0.121	0.024	0.021	0.002	0.002	0.129	0.003	0.112	0.019
	**Total**	0.658	0.292	0.009	0.325	0.241	0.124	0.266	0.177	0.314	0.018	0.058	0.085	0.009	0.000
**Hyp**	**Free**	0.027	0.020	0.253	0.031	0.000	0.070	0.000	0.001	0.004	0.044	0.092	0.049	0.080	0.031
	**Total**	0.356	0.140	0.050	0.232	0.077	0.154	0.091	0.044	0.398	0.084	0.205	0.011	0.089	0.027
**Leu**	**Free**	0.033	0.108	0.266	0.100	0.000	0.179	0.000	0.000	0.887	0.080	0.646	0.449	0.683	0.869
	**Total**	0.114	0.077	0.264	0.003	0.080	0.088	0.070	0.105	0.679	0.114	0.335	0.040	0.430	0.222
**Ile**	**Free**	0.190	0.044	0.215	0.049	0.013	0.157	0.013	0.014	0.048	0.000	0.051	0.080	0.026	0.067

	**Total**	0.031	0.253	0.180	0.079	0.264	0.191	0.263	0.264	0.134	0.129	0.147	0.064	0.152	0.281
**Met**	**Free**	0.549	0.026	0.143	0.015	0.000	0.011	0.000	0.003	0.052	0.020	0.060	0.163	0.048	0.001
	**Total**	0.126	0.005	0.198	0.174	0.011	0.295	0.015	0.004	0.608	0.053	0.466	0.009	0.308	0.234
**His**	**Free**	0.055	0.101	0.190	0.018	0.000	0.231	0.001	0.000	0.034	0.011	0.074	0.272	0.058	0.015
	**Total**	0.138	0.021	0.223	0.018	0.035	0.158	0.028	0.056	0.616	0.166	0.328	0.005	0.332	0.170
**Phe**	**Free**	0.373	0.035	0.167	0.162	0.061	0.004	0.051	0.091	0.011	0.321	0.231	0.020	0.238	0.082
	**Total**	0.087	0.092	0.131	0.000	0.014	0.208	0.011	0.025	0.010	0.072	0.012	0.028	0.012	0.024
**Glu**	**Free**	0.047	0.066	0.205	0.583	0.234	0.092	0.240	0.215	0.496	0.060	0.579	0.151	0.383	0.190
	**Total**	0.074	0.052	0.189	0.001	0.009	0.270	0.007	0.016	0.044	0.009	0.044	0.144	0.065	0.001
**Asp**	**Free**	0.105	0.065	0.004	0.007	0.055	0.495	0.051	0.066	0.302	0.091	0.083	0.000	0.052	0.323
	**Total**	0.205	0.129	0.037	0.107	0.102	0.462	0.110	0.082	0.075	0.025	0.022	0.005	0.014	0.055
**Cys**	**Free**	nd	nd	nd	nd	nd	nd	nd	nd	nd	nd	nd	nd	nd	nd
	**Total**	0.056	0.015	0.030	0.108	0.026	0.073	0.032	0.012	0.157	0.087	0.089	0.032	0.067	0.003
**Tyr**	**Free**	0.487	0.125	0.321	0.004	0.052	0.186	0.058	0.037	0.009	0.016	0.077	0.023	0.175	0.000
	**Total**	0.020	0.008	0.241	0.025	0.050	0.594	0.053	0.044	0.015	0.017	0.012	0.134	0.032	0.000
**Total**	**Free**	0.155	0.020	0.070	0.000	0.000	0.134	0.000	0.001	0.131	0.048	0.000	0.265	0.002	0.215
	**Total**	0.354	0.661	0.316	0.471	0.724	0.153	0.750	0.649	0.000	0.006	0.020	0.000	0.173	0.105
**Free/total ratio**		0.410	0.421	0.207	0.103	0.167	0.243	0.171	0.155	0.177	0.059	0.000	0.259	0.014	0.089

Ela – Elastase inhibition; Tyr – Tyrosinase inhibition; MIC* - lowest concentration at which bacterial growth is visually and differentially affected; MIC - Minimum inhibitory concentration; MBC - Minimum bactericidal concentration; ORAC - oxygen radical absorbance capacity value; CV – Keratinocytes (HaCaT) cell viability; CAA - cellular antioxidant activity; IL8 - anti-inflammatory effect
